# A phase I dose-escalation study of MEDI-575, a PDGFRα monoclonal antibody, in adults with advanced solid tumors

**DOI:** 10.1007/s00280-014-2567-9

**Published:** 2014-08-23

**Authors:** Carlos R. Becerra, Paul Conkling, Nicholas Vogelzang, Hilary Wu, Shengyan Hong, Rajesh Narwal, Meina Liang, Fatemeh Tavakkoli, Naimish Pandya

**Affiliations:** 1 Sammons Cancer Center, Texas Oncology P.A., 3410 Worth St., Suite 300, Dallas, TX 75246 USA; 2 Virginia Oncology Associates, Norfolk, VA USA; 3 Comprehensive Cancer Center of Nevada, Las Vegas, NV USA; 4 IU Health Central Indiana Cancer Centers, Indianapolis, IN USA; 5 MedImmune, Gaithersburg, MD USA; 6 University of Maryland, Baltimore, MD USA

**Keywords:** Receptor, Platelet, Platelet-derived growth factor alpha, Neoplasms, Clinical trial, phase I

## Abstract

**Purpose:**

The purpose of the study was to evaluate safety and determine the maximum tolerated dose (MTD) of MEDI-575, a fully human monoclonal antibody that selectively binds to platelet-derived growth factor receptor-α (PDGFRα), in patients with advanced solid tumors.

**Methods:**

This phase I multicenter, open-label, single-arm study enrolled adults in a 3 + 3 dose escalation design to receive MEDI-575 (3, 6, 9, 12, or 15 mg/kg) once weekly (QW) until toxicity or disease progression occurred. One 0.5-mg/kg dose was given before the first dose in the 3-mg/kg cohort to determine pharmacokinetics (PK) and pharmacodynamics under unsaturated conditions. After completion of dose escalation in the QW cohorts, patients were enrolled in two additional cohorts and received MEDI-575 25 or 35 mg/kg every 3 weeks (Q3W). Secondary measures included assessments of PK, immunogenicity, and antitumor activity.

**Results:**

A total of 35 patients received MEDI-575 QW (*n* = 23) or Q3W (*n* = 12). Most treatment-related adverse events were grade 1 or 2 in severity across all dose levels, with fatigue (*n* = 12) and nausea (*n* = 8) being reported most frequently. With no reports of dose-limiting toxicities (DLTs), the MTD was not reached. MEDI-575 exhibited a nonlinear PK profile and increased plasma platelet-derived growth factor-AA levels in a dose-dependent manner with limited immunogenicity. Stable disease was reported as the best tumor response in 9 of 29 evaluable patients; however, no objective responses were reported.

**Conclusion:**

Administration of MEDI-575 QW or Q3W resulted in a favorable safety profile, including a lack of DLTs, but without evidence of antitumor activity in patients with refractory solid tumors.

## Introduction

Platelet-derived growth factors (PDGFs) are peptide growth factors that stimulate cellular growth, proliferation, and differentiation, through transmembrane receptor tyrosine kinases PDGF receptor alpha (PDGFRα) and PDGF receptor beta (PDGFRβ) [1]. Inhibition of PDGFRα signaling has been used as an effective therapeutic strategy in diseases wherein such signaling is known to be important for tumor progression. For example, imatinib mesylate, a tyrosine kinase inhibitor with activity against PDGFRα, has been associated with a high response rate in patients with chronic eosinophilic leukemia [2], in which an *FIP1L1* *1*-PDGFRα fusion leads to aberrant activation of PDGFRα in eosinophils and other hematopoietic precursors [3]. Targeting PDGFRα in the stromal compartment, particularly in the lung, may have therapeutic potential for inhibition of tumor growth [4, 5].

MEDI-575 is an investigational human IgG2 kappa monoclonal antibody (mAb) that selectively binds to PDGFRα with a high degree of specificity, without cross-reacting with PDGFRβ [6]. Antibody targeting of this receptor is designed to allow for specific inhibition of the PDGFRα pathway without affecting PDGFRβ signaling, which has the potential for an improved toxicity profile compared with less specific small molecule inhibitors such as imatinib [7, 8]. We report the results of the first-in-human clinical study of MEDI-575 in adults with advanced solid tumors.

The primary objective of this study was to determine safety, maximum tolerated dose (MTD), and/or optimal biologic dose of MEDI-575 in adults with advanced solid tumors refractory to standard therapy or for which no standard therapy exists. Secondary objectives were assessment of the pharmacokinetic (PK) profile, immunogenicity, and antitumor activity of MEDI-575. Exploratory pharmacodynamic analysis included determination of levels of selected circulating soluble proteins (e.g., PDGF-AA).

## Methods

### Study design

This phase I, multicenter, open-label, single-arm, dose escalation study was conducted at five sites in the USA between January 2009 and April 2012 (ClinicalTrials.gov identifier: NCT00816400). Up to 42 patients [five cohorts of 3, 6, 9, 12, or 15 mg/kg dosed once weekly (QW) and two cohorts of 25 or 35 mg/kg dosed once every 3 weeks (Q3W) of 3–6 patients each] were planned for the dose escalation phase. Twelve patients were planned for inclusion in the dose expansion phase at doses determined from cohorts 1 through 7, with six patients treated on the QW dosing schedule and six patients treated on the Q3W dosing schedule. Upon review of the dose escalation data, patients in the dose expansion phase received either 9 mg/kg QW or 25 mg/kg Q3W.

### Patients

Patients were eligible for study inclusion if they were aged ≥18 years, had a histologically confirmed advanced solid tumor refractory to standard therapy or for which no standard therapy exists, had a life expectancy ≥12 weeks, had a Karnofsky performance status ≥60, and had adequate hematologic, kidney, and liver function. Patients in the dose expansion phase were limited to those with one of the following advanced solid tumors: non-small cell lung cancer (NSCLC), glioblastoma multiforme, ovarian cancer, or synovial sarcoma.

Key exclusion criteria included any prior anti-PDGF or PDGFR mAb therapy, concurrent or recent (within 4 weeks for chemotherapy or investigational therapy or within 6 weeks for biologic or immunologic therapies) standard or investigational cancer treatment, major surgery within 4 weeks of initiating MEDI-575, significant active infection requiring treatment, and use of systemic immunosuppressive steroid therapy. Prior radiation therapy was allowed, provided that exposure did not exceed an area of 25 % of marrow space, and toxicities from previous cancer therapies must have recovered to grade <2.

### Dose escalation

The starting dose of MEDI-575 was based on non-clinical PK, pharmacodynamics, and toxicology studies in cynomolgus monkeys [9]. Cohorts 1 through 5 received MEDI-575 QW as a 60-min intravenous (IV) infusion dosed at 3, 6, 9, 12, or 15 mg/kg on days 1, 8, and 15 of each 21-day treatment cycle until occurrence of unacceptable toxicity, documentation of disease progression, or other reasons for patient withdrawal. Cohort 1 received MEDI-575 0.5 mg/kg 1 week before the first 3-mg/kg dose to determine PK and pharmacodynamics under unsaturated conditions. If MTD was not reached at 15 mg/kg, cohorts 6 and 7 were planned to evaluate MEDI-575 dosed at 25 and 35 mg/kg Q3W as 90-min IV infusions until the occurrence of toxicity or disease progression.

For cycle 2 and beyond, it was permissible to delay a dose for up to 7 days based on the occurrence of clinically significant grade 2 events or first occurrences of grade 3 or 4 events, but three doses were required to complete each cycle. Discontinuation was required if grade ≥2 toxicities did not improve to grade ≤1 within 7 days of onset, if grade 3 or 4 toxicities occurred with no clinical benefit from MEDI-575, or upon second occurrence of grade 3 or 4 toxicities.

### Assessments

Safety was assessed after the first dose through 30 days after the last dose. MEDI-575 concentrations in serum were measured with a competitive electrochemiluminescence (ECL) assay using the Meso Scale Discovery^®^ platform. PDGF-AA protein in human plasma was quantified using the Milliplex™ MAP Human Cytokine/Chemokine Kit (Millipore Corporation, Billerica, MA) and Luminex^®^ xMAP technology platform (Luminex Corp., Austin, TX). An ECL-based bridging immunoassay, using a Meso Scale Discovery^®^ platform, was used for the qualitative determination of antidrug antibodies against MEDI-575 in human serum. Assessment of antitumor activity included physical examination and radiography. Tumor measurements and assessments, which were based on Response Evaluation Criteria in Solid Tumors guidelines version 1.0 (RECIST), were repeated at least 4 weeks after initial documentation of a complete or partial response.

### Statistical analyses

The safety population, used for the evaluation of baseline characteristics and safety, included all patients who received ≥1 dose of MEDI-575. The efficacy-evaluable population included patients who received ≥1 dose of MEDI-575 and had at least one tumor assessment after dosing. The MTD-evaluable population included patients who received at least one full cycle of MEDI-575 and either experienced a dose-limiting toxicity (DLT) or completed the safety follow-up through the DLT evaluation period [28 days (cohort 1) or 21 days (cohorts 2–7)] after first dose of MEDI-575.

Continuous and categorical variables for all reported outcomes were summarized by descriptive methods. Missing data were not imputed. Time to event data, including time to progression (TTP), progression-free survival (PFS), and overall survival (OS), were evaluated using Kaplan–Meier methods.

Pharmacokinetic parameters were estimated by non-compartmental analysis approach using WinNonlin Professional [version 5.2; Pharsight (Certara), Sunnyvale, CA]. Peak (maximum) concentration (*C*_max_), time to peak concentration (*T*_max_), trough serum concentration (*C*_trough_), and area under the concentration–time curve over the dosing interval (AUC_τ_) were determined after the first dose. Steady-state PK parameters, including peak concentration (*C*_max,ss_) and trough concentration (*C*_trough,ss_), were also estimated. The relationship between PK and pharmacodynamics was evaluated using a nonlinear mixed-effects model approach using NONMEM software (version 7).

## Results

**Fig. 1:**
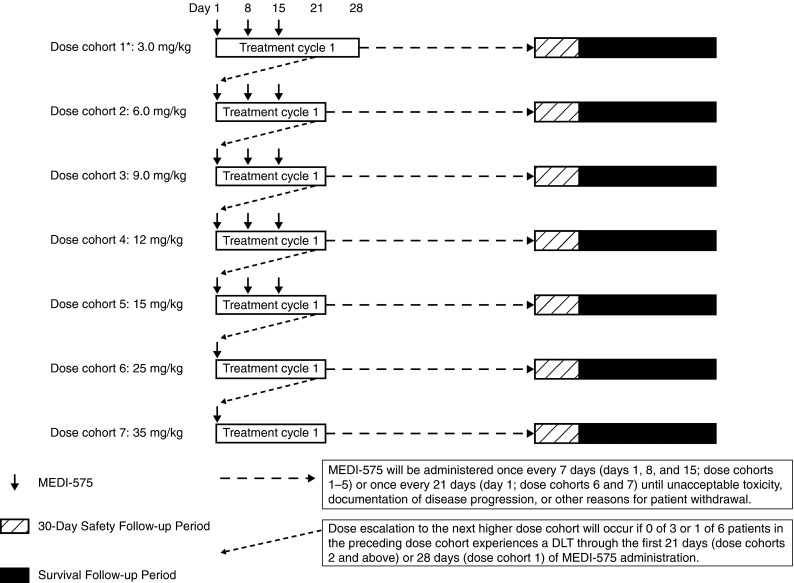
Dose escalation study design. The dose expansion phase included two additional cohorts dosed at either 9 mg/kg weekly or 25 mg/kg every 3 weeks. *Patients enrolled in dose cohort 1 received a single lead-in dose of MEDI-575 at 0.5 mg/kg on day 1 (7 days prior to receipt of the first dose of MEDI-575 3 mg/kg on day 8)

A total of 35 patients participated in the study. In the dose escalation phase, 7 cohorts (5 dosed QW and 2 dosed Q3W) received MEDI-575 QW (*n* = 17) or Q3W (*n* = 6), with doses ranging from 3 to 15 mg/kg QW or 25 to 35 mg/kg Q3W (Fig. 1). The dose expansion phase included 12 patients at 9 mg/kg QW (*n* = 6) or 25 mg/kg Q3W (*n* = 6). Overall, the patient population was predominantly white (89 %), with a median age of 65 years, baseline Karnofsky performance status of ≥80 in 92 %, and with NSCLC and colon cancer as the most common malignancies (31 and 29 %, respectively, Table 1). All patients had stage III (9 %) or IV (91 %) disease at study entry and had received a median of six prior systemic cancer treatments.

**Table 1:** Baseline demographic and clinical characteristics

Parameter, *n*	QW cohorts 1–5 (*n* = 23)	Q3W cohorts 6 and 7 (*n* = 12)	Total (*n* = 35)
Median age, years (range)	66.0 (39–83)	60.0 (50–78)	65.0 (39–83)
Sex, *n* (%)			
Male	11 (48)	8 (67)	19 (54)
Female	12 (52)	4 (33)	16 (46)
Race, *n* (%)			
White	20 (87)	11 (92)	31 (89)
Black	2 (9)	0	2 (6)
Asian	1 (4)	1 (8)	2 (6)
Ethnicity, *n* (%)			
Hispanic or Latino	3 (13)	0	3 (9)
Karnofsky performance status, *n* (%)			
60	0	1 (8)	1 (3)
70	2 (9)	0	2 (6)
80	8 (35)	4 (33)	12 (34)
90	12 (52)	7 (58)	19 (54)
100	1 (4)	0	1 (3)
Primary tumor type, *n* (%)			
Breast adenocarcinoma	1 (4)	0	1 (3)
Colon	6 (26)	4 (33)	10 (29)
Endometrial	1 (4)	0	1 (3)
Non-small cell lung	6 (26)	5 (42)	11 (31)
Ovarian	2 (9)	1 (8)	3 (9)
Prostate	2 (9)	1 (8)	3 (9)
Other	5 (22)	1 (8)	6 (17)
Stage at entry, *n* (%)			
III	3 (13)	0	3 (9)
IV	20 (87)	12 (100)	32 (91)
Median number of prior systemic treatments (range)	7.0 (2–16)	5.0 (2–13)	6.0 (2–16)

*QW* weekly, *Q3W* every 3 weeks

The median number of treatment cycles was 2 (range 1–36) across dose groups, and the median number of MEDI-575 doses received was 6 when given QW (range 1–107) and 2 when given Q3W (range 1–8).

### Safety

No DLTs were observed and the MTD was not reached for either the QW or Q3W schedules. Results showed that *C*_max_ levels of MEDI-575 above 150 µg/mL (optimal biologic concentration) were reached, starting at doses of at least 6 mg/kg. Based on emerging PK–pharmacodynamic analyses, a dose of 9 mg/kg QW and 25 mg/kg Q3W were expanded, and no DLTs were noted in patients receiving either of these doses.

Adverse events were reported in all 35 patients. The most frequently reported AEs were fatigue (54 %), nausea (34 %), vomiting (31 %), decreased appetite (26 %), dyspnea (26 %), abdominal pain, hypokalemia, insomnia, and muscle spasm (23 % each), constipation (20 %), and dehydration and diarrhea (17 % each). All treatment-related AEs by severity are presented in Table 2.

**Table 2:** All treatment-related adverse events by dose level

Event, *n*	MEDI-575 dose QW						MEDI-575 dose Q3W		
	3 mg/kg (*n* = 3)	6 mg/kg (*n* = 3)	9 mg/kg (*n* = 11)	12 mg/kg (*n* = 3)	15 mg/kg (*n* = 3)	Total (*n* = 23)	25 mg/kg (*n* = 9)	35 mg/kg (*n* = 3)	Total (*n* = 12)
Anemia	–	–	1	1	–	2	2	–	2
Asthenia	–	–	–	–	–	–	1	–	1
Blood creatinine increased	–	–	–	–	–	–	–	1	1
Chills	1	–	1	–	–	2	–	–	–
Decreased appetite	1	–	–	–	–	2	1	1	2
Dehydration	–	–	–	–	–	–	1	1	2
Diarrhea	–	–	1	–	–	1	–	1	1
Dry mouth	–	–	–	–	–	–	1	–	1
Dry skin	–	–	–	–	–	–	–	1	1
Dyspnea	–	–	1	–	–	1	–	–	–
Ear discomfort	–	–	–	1	–	1	–	–	–
Fatigue	2	1	3	1	–	7	3*	2	5*
Feeling abnormal	–	–	–	1	–	1	–	–	–
Flushing	–	–	–	–	–	–	–	1	1
Herpes zoster	–	–	–	–	–	–	1	–	1
Hypokalemia	–	–	3*	–	–	3*	2^†^	–	2^†^
Hypomagnesemia	–	–	1	–	–	1	1	1	2
Hypotension	–	–	–	–	–	–	1	–	1
Infusion reaction	1	–	–	–	–	1	–	–	–
Insomnia	–	–	–	1	–	1	–	–	–
Muscle spasms	–	–	1	2	–	3	1	–	1
Myalgia	1	1	–	–	–	2	–	–	–
Nausea	2	–	1	1	–	4	3	1	4
Peripheral edema	–	–	1	–	–	1	–	–	–
Pulmonary embolism	–	–	1^‡^	–	–	1^‡^	–	–	–
Thrombocytopenia	–	–	–	–	–	–	1	–	1
Urine urobilinogen increased	–	–	–	–	–	–	1	–	1
Vomiting	1	–	1	–	–	2	1	–	1

*QW* weekly; *Q3W* every 3 weeks

* 1 grade 3 event

^†^2 grade 3 events

^‡^1 grade 4 event

Overall, a total of 84 treatment-related AEs were reported across 24 patients: 50 (60 %) grade 1 events in 13 patients (including 1 patient at 9 mg/kg QW with peripheral edema); 28 (33 %) grade 2 events in 7 patients; 5 (6 %) grade 3 events (including 1 patient with hypokalemia at 9 mg/kg QW, 1 patient with fatigue and 2 with hypokalemia at 25 mg/kg Q3W); and 1 (1 %) grade 4 event of pulmonary embolism at 9 mg/kg QW. The most frequently reported treatment-related AEs were fatigue (reported in 7 patients in the 3 to 12 mg/kg QW groups; 5 patients in the 25 and 35 mg/kg Q3W groups) and nausea (reported in 4 patients in the 3 to 15 mg/kg QW groups; 4 patients in the 25 and 35 mg/kg Q3W groups).

Eight of 35 patients (23 %) discontinued MEDI-575 due to an AE; 1 patient at the 6-mg/kg dose with diarrhea, 4 patients at the 9-mg/kg dose (5 events: central nervous system metastasis, pericardial effusion, cerebrovascular accident, increased alkaline phosphatase, and femur fracture), and 3 patients at the 25-mg/kg dose [4 events: dyspnea, hypercalcemia (2 events in 1 patient), and NSCLC]. No discontinuations were considered related to MEDI-575.

A total of 23 serious AEs were reported in 13 of 35 (37 %) patients (Table 3). One serious AE was considered treatment-related; the aforementioned pulmonary embolism in a patient with NSCLC treated at the 9-mg/kg QW dose level. The event was reported 14 days after the first dose of MEDI-575 and caused an interruption in dosing. The patient recovered with sequelae and received a total of eight doses before discontinuing the study due to disease progression. By the end of the study, 29 deaths had occurred, 3 during treatment. All deaths were attributable to disease progression.

**Table 3:** All serious adverse events by dose level

Event, *n*	MEDI-575 dose QW						MEDI-575 dose Q3W		
	3 mg/kg QW (*n* = 3)	6 mg/kg QW (*n* = 3)	9 mg/kg QW (*n* = 11)	12 mg/kg QW (*n* = 3)	15 mg/kg QW (*n* = 3)	Total QW (*n* = 23)	25 mg/kg Q3W (*n* = 9)	35 mg/kg Q3W (*n* = 3)	Total Q3W (*n* = 12)
Total number of events	–	1	15	–	–	16	2	5	7
Total patients reporting ≥1 event	–	1	8	–	–	9	2	2	4
Pericardial effusion	–	–	1	–	–	1	–	–	–
Abdominal pain	–	–	1	–	–	1	–	–	–
Gastritis	–	–	–	–	–	–	–	1	1
Pancreatitis	–	1	–	–	–	1	–	–	–
Multi-organ failure	–	–	–	–	–	–	–	1	1
Non-cardiac chest pain	–	–	1	–	–	1	–	–	–
Cellulitis	–	–	–	–	–	–	–	1	1
Femoral neck fracture	–	–	1	–	–	1	–	–	–
Hyperglycemia	–	–	1	–	–	1	–	–	–
Fistula	–	–	–	–	–	–	–	1	1
CNS metastases	–	–	1	–	–	1	–	–	–
NSCLC	–	–	3	–	–	3	1	–	1
Cerebrovascular accident	–	–	1	–	–	1	–	–	–
Convulsion	–	–	–	–	–	–	–	1	1
Confusional state	–	–	1	–	–	1	–	–	–
Dyspnea	–	–	–	–	–	–	1	–	1
Pleural effusion	–	–	1	–	–	1	–	–	–
Pneumothorax	–	–	2	–	–	2	–	–	–
Pulmonary embolism	–	–	1	–	–	1	–	–	–

*CNS* central nervous system, *NSCLC* non-small cell lung cancer, *QW* weekly, *Q3W* every 3 weeks

### Pharmacokinetics

**Fig. 2:**
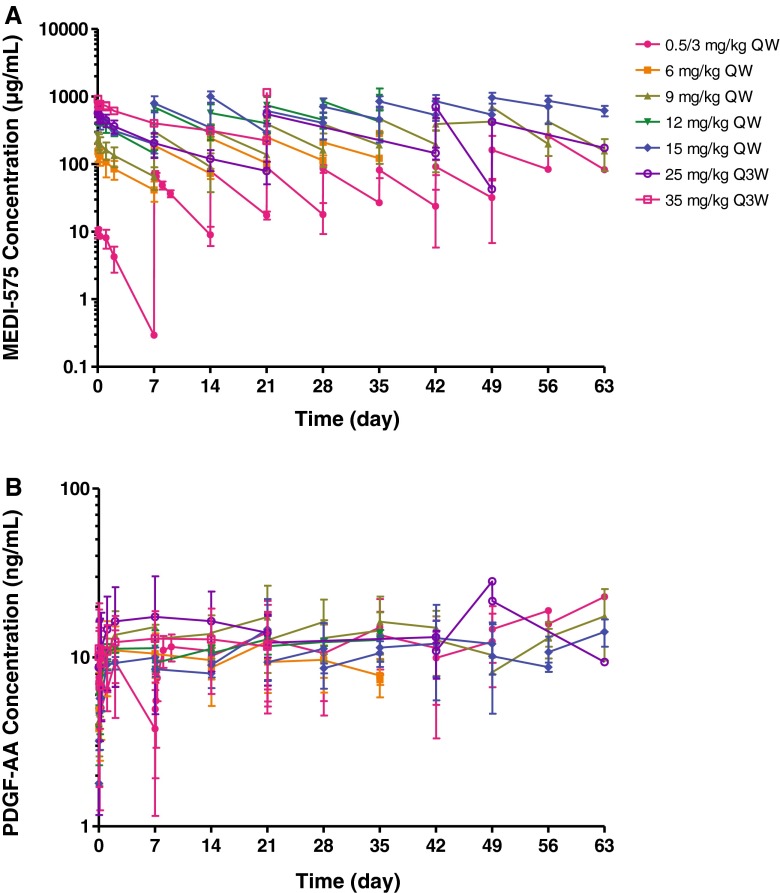
Pharmacokinetic and pharmacodynamic effects of MEDI-575. Mean **a** MEDI-575 serum concentration–time profiles and **b** PDGF-AA plasma concentration–time profiles following weekly and every 3 week dosing regimens of MEDI-575. *PDGF* platelet-derived growth factor, *QW* once weekly; *Q3W* every 3 weeks

The mean serum concentration–time profiles of MEDI-575 are shown in Fig. 2a and estimated PK parameters in Table 4. Mean serum concentrations increased with an increase in MEDI-575 dose following both QW and Q3W dosing regimens. After the first QW dose, the increase in mean AUC_τ_ (days 1–8) was more than dose proportional, reflecting nonlinearity in the PK of MEDI-575. However, mean AUC_τ_ (days 1–21) increased in an approximately dose-proportional manner after the first Q3W dose of 25 and 35 mg/kg, reflecting saturation of nonlinear elimination pathways. The *C*_max_ after the first dose increased in an approximately dose-proportional manner over the dose range of 0.5/3 mg/kg to 35 mg/kg. At steady state, the PK exposure increased in an approximately dose-proportional manner over the dose range of 3 mg/kg to 35 mg/kg. The steady-state PK exposure was similar between the 12 and 15-mg/kg QW cohorts, likely due to higher mean body weight for patients in the 12-mg/kg cohort compared with the 15-mg/kg cohort (77 vs. 68 kg). The mean linear clearance and steady-state volume of distribution were 168 mL/day and 4.42 L, respectively. The concentration corresponding to half maximum capacity (K_M_) for nonlinear clearance was 8.64 μg/mL.

**Table 4:** Mean pharmacokinetic parameters of MEDI-575

Dose (mg/kg)	*T*_max_ (day)	*C*_max_ (μg/mL)	*C*_trough_ (μg/mL)	AUC_τ_ (μg day/mL)	*C*_max,ss_ (μg/mL)	*C*_trough,ss_ (μg/mL)
0.5	0.103 (48.3)	10.9 (4.7)	BLQ (ND)	25.3 (30.4)	ND (ND)	ND (ND)
3 QW	0.076 (71.8)	72.6 (9.6)	8.97 (31.7)	201 (6.2)	104 (20.4)	35.5 (44.5)
6 QW	0.048 (6.6)	154 (29.2)	41.5 (33.2)	524 (31.7)	287 (4.9)	144 (25.6)
9 QW	0.104 (71.0)	239 (29.0)	63.1 (39.3)	874 (46.7)	387 (39.1)	202 (97.1)
12 QW	0.130 (109.3)	590 (21.1)	144 (12.5)	1,870 (14.8)	970 (36.0)	415 (46.5)
15 QW	0.107 (35.8)	632 (3.2)	199 (27.8)	2,080 (9.8)	856 (3.3)	266 (ND)
25 Q3W	0.154 (75.5)	590 (24.4)	74.6 (48.8)	4,200 (20.9)	602 (35.8)	117 (47.8)
35 Q3W	0.064 (2.2)	918 (12.1)	223 (11.3)	8,340 (4.5)	1,160 (11.8)	159 (ND)

Values are presented as mean (standard deviation)

*AUC*_*τ*_ area under the concentration–time curve, *BLQ* below the limit of quantification, *C*_*max*_ peak concentration, *C*_*max,ss*_ peak concentration at steady state, *C*_*trough*_ trough serum concentration, *C*_*trough,ss*_ trough concentration at steady state, *ND* not determined, *QW* weekly, *Q3W* every 3 weeks, *T*_*max*_ time to peak concentration

### Pharmacodynamics

A dose-dependent increase in plasma concentrations of PDGF-AA ligand was observed following IV administration of MEDI-575 QW and Q3W, consistent with the dose-dependent inhibition of PDGF-AA binding to PDGFRα and subsequent target-mediated degradation (Fig. 2b). Following the lead-in dose of 0.5 mg/kg, PDGF-AA levels increased up to 2 days, followed by a decrease to baseline levels on day 7 due to a decline in MEDI-575 concentrations to levels below the limit of quantification (BLQ). At doses higher than 3 mg/kg, the increase in plasma PDGF-AA ligand concentrations plateaued within approximately 2 days and the concentrations are sustained throughout the dosing interval. The PK–pharmacodynamic analysis was performed to describe the relationship between MEDI-575 concentrations and PDGF-AA ligand. The half-maximal concentration (IC_50_) of MEDI-575 for PDGF-AA accumulation was approximately 1.5 μg/mL. Based on the mean IC_50_, greater than 99 % saturation of PDGFRα is expected at about 150 μg/mL of MEDI-575, which can be achieved with MEDI-575 9 mg/kg QW and 25 mg/kg Q3W.

### Immunogenicity

Antidrug antibodies were detected in two patients in the 25-mg/kg Q3W cohort prior to the first administration of MEDI-575 on day 1 and were deemed false-positive results. Following the administration of MEDI-575 QW, antidrug antibodies were detected in one patient 30 days post-treatment after receiving one dose of MEDI-575 at 0.5 mg/kg followed by six doses at 3 mg/kg. No adverse events associated with the antibodies were observed. Low antidrug antibody titers (≤39–78) were observed. No obvious impact of antidrug antibodies on the PK and pharmacodynamic profiles of MEDI-575 were noted.

### Antitumor activity

No objective responses based on RECIST (v1.0) were documented. The best overall response of stable disease (SD) occurred in 9 of 29 evaluable patients (31 %), including 6 patients in the QW cohorts (1 of 3 at 3 mg/kg; 1 of 3 at 6 mg/kg; 2 of 7 at 9 mg/kg; and 2 of 3 at 15 mg/kg) and 3 patients in the Q3W cohorts (3 of 7 at 25 mg/kg). Overall, for the 29 evaluable patients, median TTP and PFS were 1.4 months (95 % CI 1.4, 1.5 months) and median OS was 8.4 months (95 % CI 3.6, 10.5 months). Median TTP and PFS were identical between the combined QW and Q3W cohorts, in whom the median OS was 7.4 months (95 % CI 3.6, 19.4 months) and 8.6 months (95 % CI 2.4, 10.5 months), respectively.

## Discussion

In this phase I study of MEDI-575 in patients with previously treated advanced solid tumors, dosing up to 15 mg/kg QW and 35 mg/kg Q3W resulted in treatment-related AEs that were predominantly grade 1 or 2 in severity. The MTD was not reached. Clinical PK and pharmacodynamic analyses identified more than 99 % PDGFRα saturation at 150 μg/mL of MEDI-575, and there was minimal evidence of immunogenicity per detectable ADAs. Stable disease was the best tumor response, with no patients achieving an objective response.

MEDI-575 is a human mAb that selectively binds to PDGFRα with high affinity, inhibiting signaling from PDGFRα on cancer cells and supportive stroma without inhibiting PDGFRβ [6]. This mechanism has important implications from a safety and tolerability standpoint, as it is recognized that inhibitors targeting both PDGFRα and PDGFRβ can lead to extravascular fluid accumulation, likely a consequence of inhibiting PDGFRβ [7]. With MEDI-575, there was only one report of treatment-related grade 1 peripheral edema at the 9-mg/kg dose level. Most treatment-related AEs did not exceed grade 2, the exceptions being 3 reports of grade 3 hypokalemia as well as individual reports of grade 3 fatigue and grade 4 pulmonary thromboembolism. The overall favorable safety profile across all MEDI-575 doses is noteworthy, especially considering the number of prior treatment regimens patients in this study population received.

Additional data to support the safety and tolerability of targeting PDGFRα in advanced malignancies are available from a phase 1 study of an anti-PDGFRα IgG1 mAb, olaratumab (formerly IMC-3G3) [10]. In that study, patients received 1 of 3 doses of antibody (4, 8, or 16 mg/kg) QW or 15 or 20 mg/kg once every 2 weeks. No DLTs or grade ≥3 AEs were observed; however, as in our study, no objective tumor responses were reported [11]. To date, for both MEDI-575 and olaratumab, preclinical observations supporting antitumor activity against lung tumors [6, 12] have not translated into advances in the clinic.

Overall, MEDI-575 exhibited nonlinear PK over the dose range of 0.5–35 mg/kg, which is consistent with receptor-mediated clearance with saturation of PDGFRα at doses of 3 mg/kg or higher. The systemic linear clearance (≈170 mL/day) and small steady-state volume of distribution (≈4 L) are in line with other mAbs [13–15]. MEDI-575 binding with PDGFRα resulted in a dose-dependent increase in PDGF-AA ligand with plateau levels within 2 days at doses ≥3 mg/kg and the concentrations are sustained throughout the dosing interval. Complete target saturation is expected at about 150 μg/mL of MEDI-575. A low incidence of immunogenicity was observed, with no resultant impact on MEDI-575 PK and pharmacodynamic parameters.

The results of this study should be considered in the context of several limitations inherent to most open-label (non-blinded) phase I studies, which are typically conducted in a small number of patients who have received multiple previous treatments and have refractory advanced disease. Most of the cohorts in this study were limited to three patients each.

In conclusion, MEDI-575 produced a favorable safety profile (including no DLTs) when administered QW or Q3W, but with no evidence of antitumor activity among adults with unselected refractory solid tumors who underwent a median of six prior systemic cancer treatments. The next step would be to identify tumors that selectively activate the pathway to determine whether blocking the signaling of the pathway with MEDI-575 may have a therapeutic effect.
